# Comparative Anti-Inflammatory Effects of Green and Red Lentil Polyphenols on Murine Dendritic Cells

**DOI:** 10.3390/foods15203738

**Published:** 2026-10-20

**Authors:** Alessandro Miraglia, Aurelia Scarano, Barbara Laddomada, Marcello Chieppa, Angelo Santino

**Affiliations:** 1 Department of Experimental Medicine (DiMeS), University of Salento, Via Monteroni, SNC, 73100 Lecce, Italy; alessandro.miraglia@unisalento.it (A.M.); marcello.chieppa@unisalento.it (M.C.); 2 Institute of Sciences of Food Production, National Research Council (CNR ISPA), Via Monteroni, SNC, 73100 Lecce, Italy; aurelia.scarano@cnr.it (A.S.); angelo.santino@cnr.it (A.S.); 3 Institute of Sciences of Food Production, National Research Council (CNR ISPA), Via Amendola, 122/O, 70126 Bari, Italy

**Keywords:** bioactive compounds, antioxidants, cytokines, nutrition, functional foods

## Abstract

Lentils are highly nutritious, polyphenol-rich pulses with potential functional properties. Here, we investigated the anti-inflammatory effects of polyphenolic extracts obtained from green and red lentil extracts (GLE and RLE) on murine bone marrow-derived dendritic cells (DCs) serving as sentinels of the immune system. GLE and RLE were analyzed for their total polyphenol content, polyphenolic profile and antioxidant activity. Our analysis revealed that GLE and RLE had comparable total content of polyphenols and similar high antioxidant capacity, but different polyphenol profiles. GLE was characterized by high levels of catechin and gallocatechin than RLE, while RLE showed higher kaempferol, myrtillin, rutin and rhoifolin content. The polyphenolic extracts were subsequently evaluated for their biological effects on immature DCs exposed to lipopolysaccharide (LPS) inflammatory stimulus. RLE induced a dose-dependent reduction in IL-1α, IL-1β and IL-6 levels similarly to quercetin, used as positive control. GLE also resulted in a dose-dependent reduction in IL-1β and IL-6 levels, however these effects were associated with marked cytotoxicity. Gene expression analysis revealed a significant reduction of IL-1α and IL-1β transcription occurring in LPS-stimulated DCs treated with 25 μM GAE of RLE, without inducing inflammatory activation in unstimulated cells. Our results demonstrate that red lentils are good source of bioactive compounds with immunomodulatory activities with enhanced anti-inflammatory activity alongside reduced cytotoxicity.

## 1. Introduction

Lentil seeds are characterized by a distinct lens-shaped morphology, exhibiting a diverse spectrum of seed coat and cotyledon pigmentations [1,2]. Commercial classification of lentils relies chiefly on seed morphology, dividing the market into two main types that include red varieties (determined by post-decortication cotyledon color) and green varieties (determined by seed coat color) [3]. Differences in the types and amounts of polyphenolic compounds between the lentil seed coat (hull) and the cotyledon drive the overall pigmentation, background color, and antioxidant capacity of lentil seeds [4].

Significant genetic variation for polyphenol content and composition has been documented across lentil germplasm including landraces, local varieties and new breeding elite lines [4,5]. A higher accumulation of flavonoids characterizes the outer seed coat, while phenolic acids were found more concentrated in the inner cotyledons [6,7,8,9]. Polyphenols not only influence lentil pigmentation, but also impact on their flavor, commercial value, marketability, and sensory properties [10,11,12]. As a result, research on lentil polyphenols have considerably increased over the last two decades, also because of their protecting role against degenerative diseases [13,14,15].

Polyphenolic extracts from lentils demonstrated significant antioxidant activity, strengthening the evidence that these pulses are a valuable source of functional compounds to prevent and manage non-communicable diseases (NCDs) [16,17]. In addition, it has been claimed that lentil extracts have a variety of pharmacological effects, including antidiabetic, hypotensive, hypolipidemic, and cardioprotective effects as they were shown through vivo and in vitro studies [13]. Previous reports showed that phenolic extract from black lentils have hepatoprotective properties by triggering the cellular antioxidant system [14], while red lentil phenolic extracts reduced sodium arsenite-induced oxidative hepatotoxicity in rats [18]. However, there is limited literature on how phenolic extracts from green and red lentils affect dendritic cells (DCs).

DCs are specialized bone marrow-derived leukocytes that serve as the primary sentinels of the immune system, acting in the human peripheral tissues, such as the skin, lungs, and gut, to capture, processing, and presenting antigens, and migrate to lymphoid organs to prime naive T cells [19]. There is evidence that polyphenols are involved in the differentiation of DCs [20,21]. Also, anthocyanins were suggested to modulate the immune system by influencing key immune cells, such as lymphocytes, macrophages, and dendritic cells, while maintaining a balance between proinflammatory and anti-inflammatory cytokines [22,23].

In the present study, we investigated the polyphenolic composition and the antioxidant capacity of extracts from green (GLE) and red lentils (RLE). In addition, we evaluated the potential ability of GLE and RLE to modulate immune responses and reduce inflammation-related markers through their effect on dendritic cells. We hypothesized that the treatment with RLE would be most effective at reducing inflammation due to a different polyphenolic composition compared to that of GLE. The results contribute to support the potential role of red lentils as valuable functional foods and sources of bioactive compounds with antioxidant and anti-inflammatory properties.

## 2. Materials and Methods

### 2.1. Plant Material

Two lentil genotypes were used in the present study differing for seed color and size, namely a red lentil variety with small seed (IG1117646), and an elite line with green and large seeds (6002/ILWL118/1-1) both received from International Center for Agricultural Research in the Dry Areas (ICARDA) and grown at ICARDA Marchouch experimental station (33.56° N, 6.63° W, 392 m altitude) in Morocco. A randomized complete block design with three blocks was used to account for potential environmental variation affecting phenolic acid accumulation in the grain [4]. The three biological replicates were analyzed separately in duplicate (two technical replicates) for polyphenol profile and antioxidant activity to evaluate variability among the experimental blocks. Because no significant differences were detected, the three biological replicates for both GLE and RLE were pooled into a single composite sample for subsequent assays on murine cells.

### 2.2. Extraction and Quantification of Total Polyphenols (TP)

Total polyphenols were extracted from whole-meal flour samples obtained by milling whole grain seeds using a Mixer Mill (MM 400 Retsch, Verder Scientific S.r.l. Pedrengo, Italy) with a frequency of 30 Hz for 1 min. A volume of 1 mL methanol/water/formic acid (80/15/5) was added to the whole-meal flour (0.1 g), and the mixture was incubated at room temperature overnight. Samples were centrifuged at 4000× *g* for 10 min, and the slurry was re-extracted for a second time adding 1 mL of same solvent on a rotary shaker for 1 h [7]. The two supernatants were collected in one tube. Total polyphenol content was measured according to the protocol described by [24]. Gallic acid was used to obtain a calibration curve in the range from 2.5 to 40.0 mg/L. The total phenol content of each sample was expressed as gallic acid equivalents (GAE). The analyses were carried out in triplicate.

### 2.3. HPLC Analysis

Individual phenolic compounds were identified and quantified using an Agilent-1100 liquid chromatograph (Agilent Technologies, Santa Clara, CA, USA) equipped with a DAD detector (Agilent 1260 Infinity), and a C18 column (5 µm UltraSphere 80 Å, 4.6 internal diameter × 250 mm length) according to details previously described [7,25]. Results were expressed as micrograms per gram of dried matter. For calibration curves, flavonoid standards were purchased from Sigma-Aldrich (Milan, Italy).

### 2.4. Determination of Antioxidant Capacities of TP Extracts

TPC extracts were evaluated for antioxidant capacity according to the protocol by [26]. The absorbance of samples was read at 750 nm, and the antioxidant capacity was expressed as mmol Trolox equivalent antioxidant capacity (TEAC) per g dry matter.

### 2.5. Animals, Culture and Treatment of Murine BMDCs

All animal procedures were conducted in accordance with protocol guidelines approved by the Institutional Animal Care Committee of the University of Salento (Animal Welfare Body, OPBA). Experiments complied with Italian Legislative Decree No. 26/2014 and received approval from the Italian Ministry of Health—General Directorate of Animal Health and Veterinary Drugs (DGSAF, Protocol No. 98/2024-PR, dated 23 January 2024) in accordance with the 3Rs principles. The animals were fed “ad libitum” and housed under standard environmental conditions, with a regular day-night cycle (light: 08:00–20:00), an ambient temperature of 20–22 °C, and humidity between 45% and 55%, with daily monitoring of animal welfare by dedicated personnel. Animals were humanely euthanized by cranio-cervical dissociation at 16 weeks of age; this procedure was carried out strictly post-mortem by highly qualified personnel. No humane endpoints were applied to this specific cohort. Bone marrow-derived dendritic cells (BMDCs) were generated from the tibiae and femurs of three 16-week-old male C57BL/6 wild-type (WT) mice from the control-diet group. These tissues were harvested after completing the primary study protocol. BMDCs were preferred over immortalized cell lines, as the latter often exhibit genetic aberrations and fail to faithfully replicate the physiological maturation and cytokine profiles of functional dendritic cells. Once harvested, the bone marrow was washed with PBS containing 0.5 mM EDTA (Thermo Fisher Scientific, Waltham, MA, USA). The collected material was centrifuged at 1750 rpm at 4 °C for 10 min. The supernatant was discarded, and the resulting cell pellet was deprived of red blood cells by treatment with ACK (ammonium chloride potassium) lysis buffer (Thermo Fisher Scientific, Waltham, MA, USA). The resulting solution was centrifuged a second time following the above conditions to remove residual red blood cells. Cell pellet was resuspended in RPMI-1640 medium (Thermo Fisher Scientific, Waltham, MA, USA), supplemented with 10% heat-inactivated fetal bovine serum (FBS) (Thermo Fisher Scientific, Waltham, MA, USA), 2 mM L-glutamine, 1 mM sodium pyruvate, 1 mM non-essential amino acids, 25 mM HEPES, 100 U mL^−1^ penicillin/streptomycin (Thermo Fisher Scientific, Waltham, MA, USA), 25 ng/mL mGM-CSF (Miltenyi Biotec, Bergisch Gladbach, Germany), and 25 ng/mL mIL-4 (Miltenyi Biotec, Bergisch Gladbach, Germany). The cells were maintained in culture for 10 days, with intermediate passages on days 3 and 7, and then re-stimulated with new growth factors [27]. On day 10, dendritic cells were purified using the EasySep™ Mouse CD11c Positive Selection Kit (Stemcell Technologies, Vancouver, NA, Canada). The immature dendritic cells (BMDCs) coming from single animal were seeded in equal numbers, treated, and incubated with scalar concentrations (6 μM, 12 μM, and 25 μM Gallic Acid Equivalents, GAE) of the two tested lentil polyphenolic extracts, green and red lentils from the elite lentil 6002/ILWL118/1-1 line, respectively. The procedure was repeated for each biological replicate (n = 3). The methanolic extracts used for cell treatment were the same obtained for chemical analyses, sterilized by filtration and directly diluted in cell culture medium to obtain the desired final GAE concentrations. GAE values were used to express the content of total polyphenols administered to the cells, based on the total polyphenol content determined for each methanolic extract. Quercetin was used as a positive control at final concentration of 25 μM. A methanol control was also included to assess any effects attributable to the extraction solvent, in the same final concentration as that present in the corresponding extract-treated cultures. After 24 h of treatments, the cells were stimulated with 1 μg/ml of Salmonella Typhimurium lipopolysaccharide (LPS) (Sigma-Aldrich, St. Louis, MO, USA). After 48 h, the supernatants were collected and used to evaluate the expression of secreted cytokines.

### 2.6. Cell Viability Test

Cell viability was evaluated by using the MTT assay. At the end of treatment, 0.5 mg/ml MTT was added to the culture medium for each experimental condition and incubated for 3 h. The culture medium was then removed, and the formazan crystals were solubilized in 0.04 N isopropanol/HCl. The lysates were subsequently read spectrophotometrically at 550 nm (Bio-rad, Richmond, CA, USA). The results were expressed as percentage viability compared to the control [28].

### 2.7. ELISA Test

The quantification of the cytokines IL-1β, IL-1α, and IL-6 in the supernatants of cells treated with the extracts was performed using the ELISA (Enzyme-Linked ImmunoSorbent Assay) method. Briefly, a 96-well plate coated with the capture antibody overnight was blocked for at least 1 h, followed by sequential 2-h incubations with samples/standards and the detection antibody. The plate was then incubated with Streptavidin-HRP for 20 min and developed with Substrate Solution for another 20 min in the dark, with all steps performed at room temperature. The analysis was conducted using commercial kits from R&D Systems^®^ (Minneapolis, MN, USA)according to the manufacturer’s recommended protocols [29].

### 2.8. Gene Expression Analysis by Real-Time PCR

Total RNA was isolated from BMDCs using TRIzol^®^ (Thermo Fisher Scientific, Waltham, MA, USA) according to the manufacturer’s instructions. Total RNA (1 µg) was reverse transcribed using an iScript cDNA Synthesis kit (Biorad, Hercules, CA, USA) with random primers for cDNA synthesis. Gene expression of IL-1β, IL-1α, TNF, and Gapdh was tested with PrimePCRTM SYBR^®^ Green Assay murine primers (qMmuCID0005641, qMmuCID0005637, qMmuCED0004141, and qMmuCED0027497—Biorad, Hercules, CA, USA, respectively). Real-time analysis was performed on a CFX96 system (Biorad Laboratories, Hercules, CA, USA) and the expression of all target genes was calculated relative to Gapdh expression using the 2^−ΔΔCt^ method [30].

### 2.9. Statistical Analysis

Overall data were expressed as mean ± SD from three independent experiments. Statistical analysis of biological data was performed using GraphPad Prism version 9.0 statistical software. We assessed statistical significance with the two-tailed Student’s *t*-test. Results were considered statistically significant at *p* < 0.05.

## 3. Results and Discussion

### 3.1. Polyphenol Composition and Antioxidant Activity

**Figure 1:**
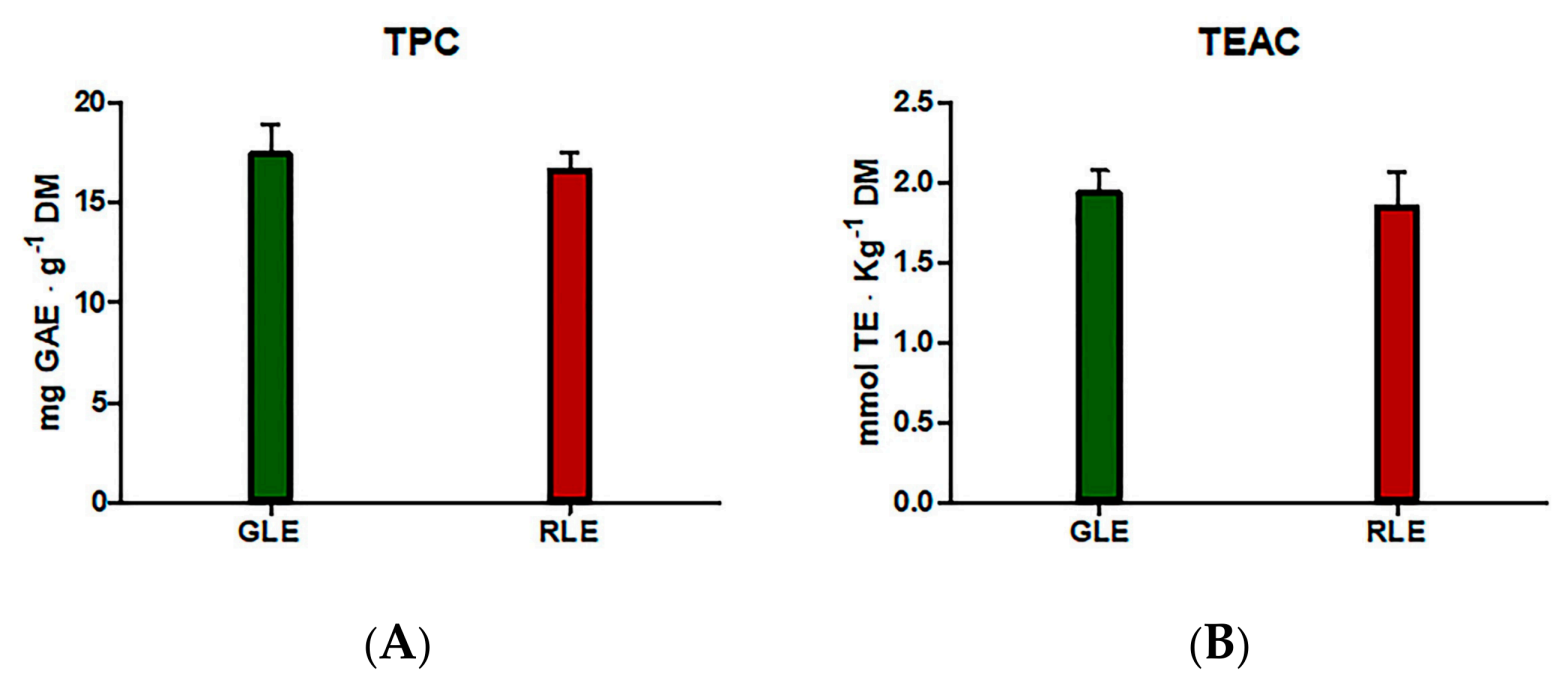
(**A**) Total polyphenol content (TPC) measured by the Folin-Ciocalteu assay, and (**B**) antioxidant activity measured using TEAC assay in green (GLE) and red (RLE) lentil methanolic extracts.

Lentil seeds contain a chemically diverse array of phenolic constituents whose abundance and distribution are strongly influenced by genotype and seed pigmentation [9,31,32]. Flavonoid-related compounds represent a major fraction of this phytochemical diversity and may contribute differently to the biological properties of individual lentil varieties. In the present study, we compared acidified methanolic extracts obtained from whole-meal flour of green and red lentils to determine whether differences in seed phenotype were associated with distinct polyphenolic profiles and antioxidant properties. Total polyphenol content (TPC) and radical-scavenging activity were therefore assessed in green (GLE) and red lentil extracts (RLE). The results shown in Figure 1 indicate that GLE had slightly higher TPC and antioxidant capacity compared to RLE. Previous works also showed that green lentils generally exhibit significantly higher TPC and antioxidant capacity than red lentils [33]. This is due because green lentils are consumed and analyzed as whole seeds with their nutrient-rich, pigmented seed coats intact [33]. Conversely, red lentils are usually decorticated for consumption and often analyzed as split lentils which notably causes a loss of many phenolic-rich layers [34,35].

**Figure 2:**
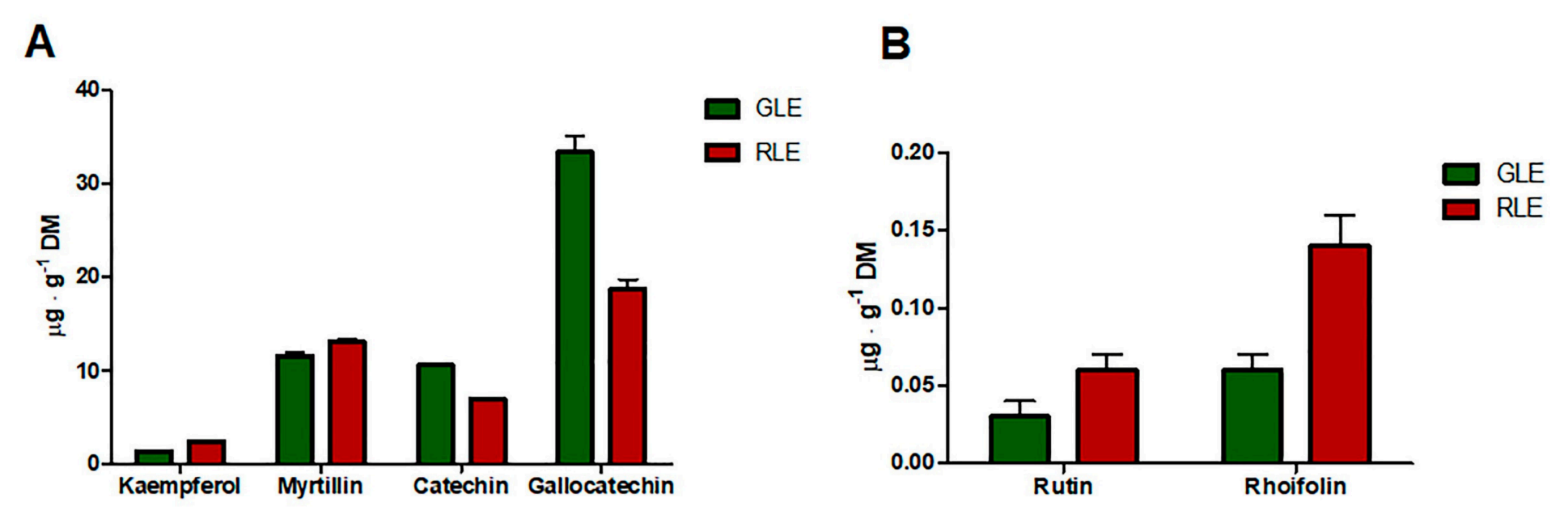
(**A**) Kaempferol, myrtillin, catechin and gallocatechin contents measured by RP-HPLC analysis. (**B**) Rutin and rhoifolin content measured by RP-HPLC analysis in green (GLE) and red (RLE) lentil methanolic extracts.

In this study, TPC reflected the occurrence of polyphenols in whole seeds, resulting in 16.59 mg GAE g^−1^ in RLE and 17.42 mg GAE g^−1^ in GLE (Figure 1A) which are comparable to previous works [31,36,37]. We also evaluated the antioxidant activity of TPC extracts as radical scavenging capacity (ABTS) and the results are shown in Figure 1B. We found that two extracts had comparable ABTS values (1.94 TE mmol Kg^−1^, GLE; 1.85 mmol Kg^−1^, RLE). The antioxidant performance of lentil extracts cannot be attributed exclusively to their overall phenolic concentration. Differences in the molecular composition of the extracts, together with interactions among individual constituents, may substantially influence their radical-scavenging activity [38,39]. Phenolic compounds can interfere with oxidative reactions through several complementary chemical mechanisms, including electron or hydrogen transfer and interactions with redox-active metal ions [40,41]. Thus, the comparable ABTS values observed for GLE and RLE may reflect the combined contribution of compounds differing in structure and relative abundance. A total of six phenolic compounds were identified and quantified in GLE and RLE by HPLC DAD analysis (Figure 2). By considering the results across the two extracts, gallocatechin was the most abundant phenolic compound, followed by catechin, myrtillin, kaempferol, rhoifolin, and rutin (Figure 2).

The flavanol gallocatechin was the predominant compound present in both the extracts. ranging between 18.65 µg g^−1^ d.m. (RLE) and 33.35 µg g^−1^ d.m. (GLE). Also, catechin, another flavanol detected in the samples, was higher in GLE (10.53 µg g^−1^ d.m.) compared to RLE (6.98 µg g^−1^ d.m.). Conversely, myrtillin (an anthocyanin compound), and kaempferol (a flavonol), showed a higher content in RLE compared to GLE (Figure 2). The least abundant compounds, namely rhoifolin and rutin, showed higher levels in RLE (0.14 and 0.06 μg g^−1^ d.m., respectively) compared to GLE (0.06 and 0.03 μg g^−1^ d.m.). Overall, all the polyphenolic compounds detected in GLE and RLE were likely to contribute to the antioxidant potential of lentil extracts, as also reported in other previous studies [39]. Polyphenolic compounds can play a crucial role in preventing the oxidative processes stimulated by the free radicals, enhancing the nutritional properties of plant-derived foods and providing benefits for human health [22,42,43].

### 3.2. Cell Viability

To investigate the potential effects of polyphenolic lentil extracts, we initially evaluated the cell viability of murine bone-marrow dendritic cells (DCs), which were used as in vitro models in this study. DCs provide an experimental model of innate immune activation, since LPS drives them to a pro-inflammatory state with robust cytokine production, making them useful for testing anti-inflammatory compounds [40]. Cell viability was assessed after treatment with GLE and RLE methanolic extracts at different concentrations (6, 12, 25, and 50 µM gallic acid equivalents, GAE), in the presence or absence of LPS. Cell viability analysis revealed substantial differences between the two extracts. In fact, results showed that red lentil extract (RLE) did not affect dendritic cells’ viability even when administered at elevated concentration. Conversely, green lentil extracts (GLE) significantly reduced DCs viability compared to red lentil extract or vehicle.

**Figure 3:**
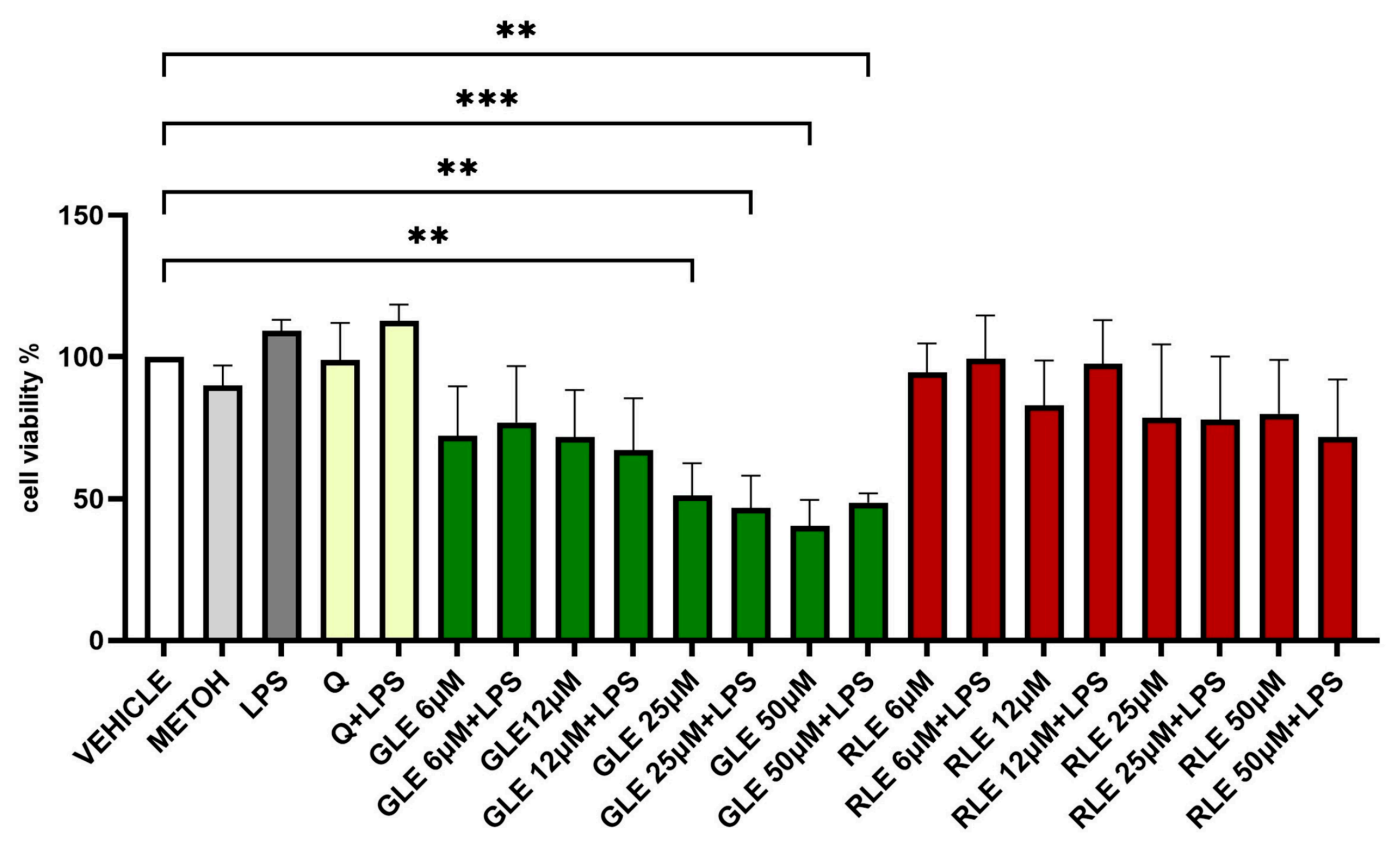
Effect of green (GLE) and red (RLE) lentil polyphenolic extracts on cell viability. Cell viability was evaluated after treatment with green and red lentil extracts at different concentrations (6, 12, 25, and 50 µM GAE) in the presence or absence of LPS administration for 48 h. Data are expressed as mean ± SD of independent experiments. Statistical significance was determined with the two-tailed Student’s *t*-test. Asterisks indicate significant differences compared with the control group (** *p* < 0.01; *** *p* < 0.001).

A dose-dependent reduction in cell viability was observed with and without LPS (Figure 3). GLE reduced DCs viability to approximately 70–75% at a concentration of 6 µM GAE. At higher concentrations, namely 25 and 50 µM GAE, DCs viability furtherly decreased, reaching values of approximately 50–65% and 40–50%, respectively. These results suggest that some compounds present in the GLE methanolic extract, e.g., catechin and gallocatechin, may exert cytotoxic effects even at low concentrations. The higher abundance of catechin and gallocatechin in GLE may contribute to the distinct cellular response observed with this extract, although a direct causal relationship cannot be established from the present data. Catechin-related compounds are generally associated with antioxidant activity; however, their effects in cellular systems vary according to experimental context, exposure level, and the specific phenolic structure considered [43,44,45].

In some cases, phenolic compounds may also promote oxidative imbalance or adversely affect cell survival. The marked reduction in DC viability observed after GLE exposure therefore suggests that the biological activity of this extract results from concentration-dependent interactions between its phytochemical components and the cellular environment. In contrast to what observed with GLE, red lentil extracts did not modify cell viability which resulted comparable to that of the control (~95–100%). This result was confirmed also using higher concentrations of RLE (12 and 25 µM GAE), with cell viability still resulting relatively high (~80–95%) (Figure 3). A moderate reduction (~70–80%) was observed using 50 µM GAE of RLE.

Overall, the results indicate that GLE exerts a pronounced, dose-dependent cytotoxic effect while RLE exhibited cellular biocompatibility under the tested experimental conditions. Such differences between the two extracts may reflect the variations in phytochemical composition among lentil varieties. In fact, although lentils are widely recognized as a rich source of bioactive compounds with potential health benefits, including polyphenols, flavonoids, and other phytochemicals, their biological effects can vary depending on their concentration composition and relative abundance [38].

### 3.3. Quantitative Determination of Cytokines by ELISA

**Figure 4:**
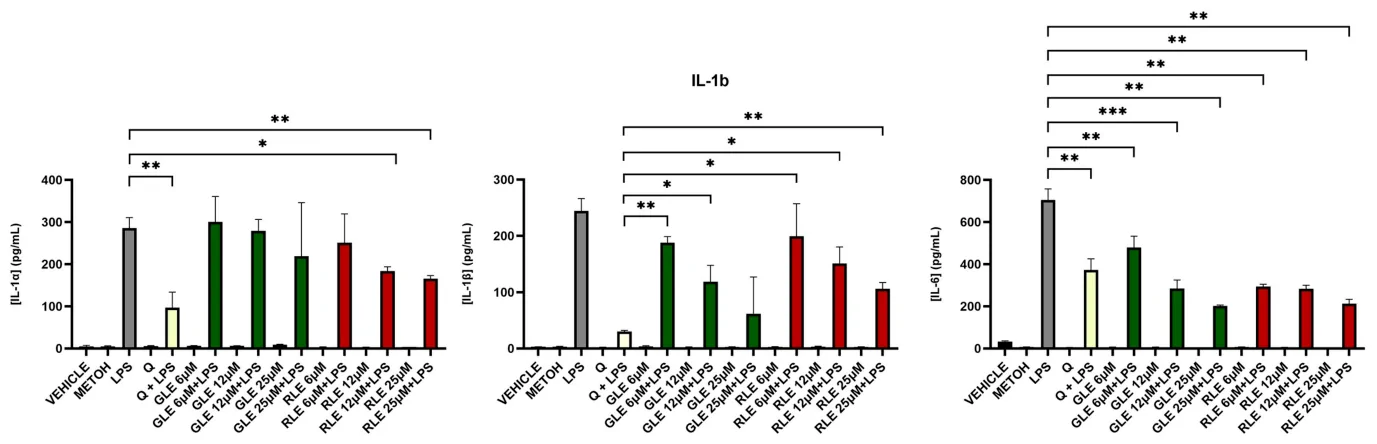
Effect of green (GLE) and red (RLE) lentil polyphenolic extracts on pro-inflammatory cytokine secretion in LPS-stimulated cells. IL-1α, IL-1β, and IL-6 concentrations (pg/mL) were quantified in cell supernatants 48 h after LPS treatment in the presence or absence of quercetin (Q) or GLE and RLE lentil extracts at different concentrations (6, 12, and 25 μM GAE). LPS treatment induced a significant increase in cytokine production compared to controls, vehicle and methanol (MetOH). Quercetin significantly reduced LPS-induced cytokine levels. Data are expressed as mean ± standard deviation (SD) of independent experiments. Statistical analysis was performed using the Student’s *t*-test. * *p* < 0.05, ** *p* < 0.01, *** *p* < 0.001.

Treatment with GLE resulted in a dose-dependent reduction in IL-1β and IL-6 levels following LPS administration, while the reduction in IL-1α was less pronounced (Figure 4). The most significant reductions occurred at concentrations associated with significant cytotoxicity. The observed anti-inflammatory potential of GLE is very likely the effect of cell necrosis due to the extract cytotoxicity. The reduction in cytokine production at higher concentrations of GLE could be attributable to the reduction in cell number rather than a specific anti-inflammatory mechanism. As cell death is a regulator of inflammation via cytokine IL-1, the release of IL1α or IL1b depends on mechanism of cell death [46]. In fact, necroptotic stimuli can induce a release of IL-1α [46], and necrotic cells have also been reported to likely release IL-1α [47]. Previous works have also shown that catechins can reduce cell viability and cytokine-induced inflammation [48]. Specifically, epigallocatechin-3-gallate has been shown to cause a concentration-dependent decrease in cell viability of LPS-stimulated BV-2 microglia cells at high concentrations and to inhibit the release of the pro-inflammatory cytokine IL-6 [49]. Therefore, the possible anti-inflammatory effects of GLE appear to be limited by its cytotoxic profile under the experimental conditions tested.

In contrast, treatment with RLE induced a dose-dependent reduction in IL-1α, IL-1β and IL-6 levels, comparable to the anti-inflammatory effect of quercetin administration used as control (Figure 4). This observation suggests that the inhibitory abilities shown by RLE on cytokine release were due to the suppression of the LPS mediated inflammatory pathway. Indeed, consistent with the expected inflammatory response, LPS stimulation significantly increased IL-1α, IL-1β, and IL-6 production, while the positive control (quercetin, Q) effectively reduced both cytokines, confirming its anti-inflammatory activity, in line with our previous results [50,51]. Quercetin dampens LPS-induced activation of murine dendritic cells and suppresses the release of pro-inflammatory mediators, resulting in reduced cytokine and chemokine production [52]. Consistent with these observations, quercetin has also been shown to decrease IL-1β and IL-6 production in LPS-stimulated immune cells [52,53].

Overall, the results suggest that RLE was more effective as anti-inflammatory extract, reducing the production of pro-inflammatory cytokines while preserving cell viability. In fact, the results suggest that RLE can promote a specific modulation of the inflammasome like other plant bioactive extracts [54]. Previous studies have reported that polyphenol-rich lentil extracts can attenuate inflammatory responses in LPS-stimulated cells by reducing the production of inflammatory mediators such as IL-6 and IL-1β [55,56]. Similarly, lentil hull polyphenols and their digestion-derived metabolites have been shown to mitigate LPS-induced inflammation through the downregulation of inflammatory cytokines. Beyond their anti-inflammatory effects, these compounds have also been reported to improve intestinal barrier integrity, highlighting their potential as functional dietary ingredients for the prevention of inflammatory diseases [55].

### 3.4. RLE Modulates Inflammatory Molecular Signatures in DCs

**Figure 5:**
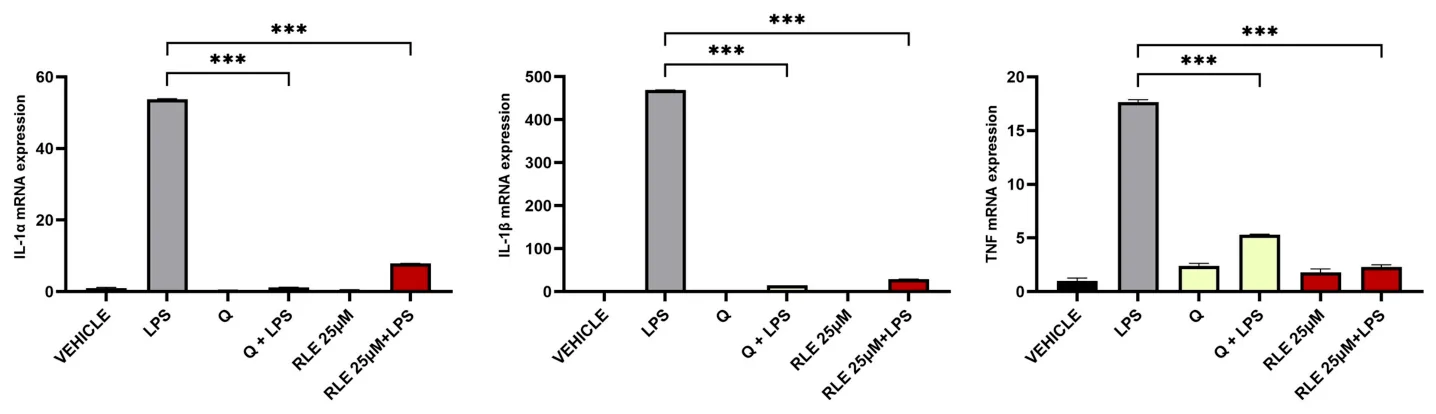
Effect of red lentil polyphenolic extract (RLE) on LPS-induced pro-inflammatory cytokine expression. Relative mRNA expression levels of IL-1α, IL-1β, and TNF were determined by RT-qPCR in cells treated with red lentil extract (25 μM GAE) in the presence or absence of lipopolysaccharide. LPS stimulation significantly increased the expression of all pro-inflammatory cytokines analyzed compared to the control group. Co-treatment with quercetin (Q) significantly suppressed LPS-induced cytokine expression. Similarly, treatment with red lentil extract and subsequent LPS significantly attenuated the upregulation of IL-1α, IL-1β, and TNF-α compared to cells treated with LPS alone. Gene expression levels were normalized to the housekeeping gene and expressed as fold change compared to control cells. Data are expressed as mean ± standard deviation (SD) of independent experiments. Statistical analysis was performed using the Student’s *t*-test. *** *p* < 0.001.

To further explore our hypothesis that RLE can efficiently promote the anti-inflammatory pathway in DCs, we analyzed the transcriptional expression of key pro-inflammatory cytokines after treatment with 25 µM GAE of RLE. Results shown in Figure 5 indicated that RLE administration did not induce the transcription of the inflammatory cytokines, suggesting that it did not trigger inflammatory activation in quiescent cells but significantly reduced the transcription of IL-1α and IL-1β after LPS stimulation. Similarly, TNF expression was remarkably reduced in DCs treated with RLE compared to those exposed to LPS alone, although the inhibitory effect of the RLE was less pronounced than that observed in presence of quercetin exposure.

Previous studies indicate that exposure to selected dietary phenolic compounds can alter dendritic-cell responses to inflammatory stimuli, resulting in lower cytokine production and changes in pathways associated with immune activation [57,58]. In parallel, lentil-derived phenolic fractions have been reported to attenuate inflammatory signaling in experimental cell systems [58].

Altogether, these results demonstrate that RLE induced an efficient reduction in IL-1α, IL-1β and IL-6 secretion, together with high cell viability. Importantly, the extract did not significantly alter basal cytokine expression when administered alone, suggesting that it does not trigger inflammatory activation in quiescent cells, and highlighting its potential role as a natural source of bioactive compounds with anti-inflammatory properties. However, the pretreatment with RLE significantly attenuated the LPS-induced upregulation of IL-1α, IL-1β, and TNF. These results indicate that RLE exposure can suppress the DCs transcription of inflammatory mediators induced by LPS. Although direct dendritic-cell data on red lentil polyphenols remain limited, recent studies on lentil-derived phenolics and related dietary polyphenols support an anti-inflammatory profile, with suppression of LPS-linked inflammatory mediators and cytokines in immune and intestinal cell models, as well as attenuation of colonic inflammation in lentil-fed mice [59,60].

## 4. Conclusions

This study shows the potential biological activities of lentil extracts, by determining the polyphenolic profile, the antioxidant capacity and the anti-inflammatory effects on DCs. Although both lentil extracts displayed a comparable high antioxidant capacity, they differed in their polyphenolic composition and biological effects. In particular, GLE showed higher cytotoxicity and less effective anti-inflammatory under our experimental conditions. RLE reduced the secretion and transcription of pro-inflammatory cytokines in LPS-stimulated DCs without inducing inflammatory activation in unstimulated cells. Importantly, RLE exhibited a more favorable balance between efficacy and cytotoxicity, supporting the potential role of red lentils as a source of bioactive compounds with immunomodulatory properties, and functional ingredient or nutraceutical for the management of inflammation.

## Data Availability

The original contributions presented in this study are included in the article material. Further inquiries can be directed to the corresponding author.
